# Bromidotris(triphenyl­phosphane)silver acetonitrile monosolvate monohydrate

**DOI:** 10.1107/S1600536811040827

**Published:** 2011-10-08

**Authors:** Anita M. Owczarzak, Loukas Kyros, Sotiris K. Hadjikakou, Maciej Kubicki

**Affiliations:** a Department of Chemistry, Adam Mickiewicz University, Grunwaldzka 6, 60-780 Poznań, Poland; b Section of Inorganic and Analytical Chemistry, Department of Chemistry, University of Ioannina, 45110 Ioannina, Greece

## Abstract

In the title compound, [AgBr(C_18_H_15_P)_3_]·C_2_H_3_N·H_2_O, the coordination of the Ag atom is close to ideal tetra­hedral, with the three Ag—P bond lengths almost equal [2.5441 (10), 2.5523 (9) and 2.5647 (10) ° A] and the Ag—Br bond slightly longer [2.7242 (5) Å]. The coordination tetra­hedron is slightly flattened, the Ag atom is closer to the PPP plane; the P—Ag—P angles are wider than the Br—Ag—P angles. The voids in the crystal structure are filled with ordered acetonitrile solvent mol­ecules. The remaining electron density was inter­preted as a water mol­ecule, disordered over three alternative positions. Neither of the solvent mol­ecules is connected by any directional specific inter­actions with the complex.

## Related literature

For general background to silver complexes and their biological activity, see: Blower & Dilworth (1987 ▶); Zartilas *et al.* (2009 ▶). For a similar complex without the solvent mol­ecules, see: Engelhardt *et al.* (1987 ▶). For a description of the Cambridge Structural Database, see: Allen (2002 ▶).
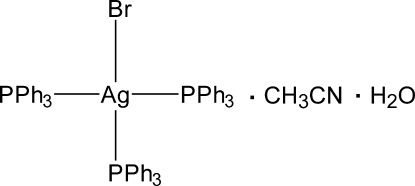


## Experimental

### 

#### Crystal data

[AgBr(C_18_H_15_P)_3_]·C_2_H_3_N·H_2_O
*M* *_r_* = 1033.66
Triclinic, 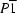

*a* = 13.1894 (4) Å
*b* = 13.7384 (5) Å
*c* = 13.8299 (5) Å
α = 84.103 (3)°
β = 87.161 (3)°
γ = 77.398 (3)°
*V* = 2431.73 (14) Å^3^
*Z* = 2
Mo *K*α radiation
μ = 1.38 mm^−1^
*T* = 100 K
0.3 × 0.3 × 0.2 mm

#### Data collection

Agilent Xcalibur Sapphire2 diffractometer
Absorption correction: multi-scan (*CrysAlis PRO*; Agilent, 2010 ▶) *T*_min_ = 0.956, *T*_max_ = 1.000
17990 measured reflections
9347 independent reflections
7605 reflections with *I* > 2σ(*I*)
*R*_int_ = 0.029

#### Refinement

*R*[*F* ^2^ > 2σ(*F* ^2^)] = 0.043
*wR*(*F* ^2^) = 0.102
*S* = 1.04
9347 reflections
591 parameters
19 restraints
H-atom parameters constrained
Δρ_max_ = 1.27 e Å^−3^
Δρ_min_ = −0.91 e Å^−3^

### 

Data collection: *CrysAlis PRO* (Agilent, 2010 ▶); cell refinement: *CrysAlis PRO*; data reduction: *CrysAlis PRO*; program(s) used to solve structure: *SIR92* (Altomare *et al.*, 1993 ▶); program(s) used to refine structure: *SHELXL97* (Sheldrick, 2008 ▶); molecular graphics: *SHELXTL* (Sheldrick, 2008 ▶) and *Mercury* (Macrae *et al.*, 2008 ▶); software used to prepare material for publication: *SHELXL97*.

## Supplementary Material

Crystal structure: contains datablock(s) I, global. DOI: 10.1107/S1600536811040827/go2030sup1.cif

Structure factors: contains datablock(s) I. DOI: 10.1107/S1600536811040827/go2030Isup2.hkl

Additional supplementary materials:  crystallographic information; 3D view; checkCIF report

## Figures and Tables

**Fig. 1.**
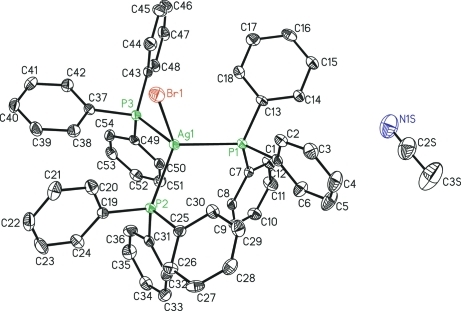
Anisotropic ellipsoid representation of molecule 1 together with atom labelling scheme. The ellipsoids are drawn at 50% probability level, hydrogen atoms are omitted for clarity.

## supplementary crystallographic information

### Comment

Silver(I) complexes adopt geometries with variable nuclearities and structural diversity (*e.g.* Blower & Dilworth, 1987), which exhibit a wide range of applications in medicine, in analytical chemistry or in industry of polymers. Recently, Ag(I) complexes have also been studied for their antitumor activity (*e.g.* Zartilas *et al.*, 2009 and references therein). This makes the study of silver(I) chemistry very attractive, since the molecular design and structural characterization of silver(I) complexes with particular properties is therefore an intriguing aspect. In this context, our research has been focused for some time on coordination compounds of silver(I) with a large range of heterocyclic thiones containing triarylphosphines as bulky *p*-acceptor co-ligands such as triphenylphosphine, whereby particular emphasis has been placed on the determination of the factors causing variations of silver(I) geometry.

In the crystal structure of the titled compound the coordination of silver atom is tetrahedral. All Ag—P bond lengths are almost equal, mean value is 2.554 (10) Å, while Ag—Br is slightly longer, of 2.7242 (5) Å. These values are on the long-bond end of the values found in the Cambridge Crystallographic Database (Allen, 2002; Version 5.32 of Nov. 2010, last update Aug. 2011); the mean values are 2.45Å for Ag—P and 2.65Å for Ag—Br (Br not bridging). The coordination tetrahedron is slightly flattened, the Ag atom is closer to the PPP plane. It might be seen also from the *X*—Ag—*X* angle pattern: all P—Ag—P angles are larger than the Br—Ag—P angles.

In the crystal structure the voids are filled with the ordered acetonitrile molecule and with a remaining electron density which was interpreted as a water molecule, disordered over three alternative positions. Due to the lack of the directional interactions the crystal packing are probably determined by Weak π···π interactions (the separation between parallel C13···C18 phenyl rings related by the center of symmetry is 3.46 Å) and some van der Waals-type dispersion interaction. The presence of the solvent - acetonitrile and the residual electron density which fills the voids (and was interpreted as the disorder water molecules causes that the complex looses its C_3_ symmetry which was reported for the unsolvated structure (Engelhardt *et al.*, 1987).

### Experimental

All solvents used were of reagent grade, while silver bromide, triphneylphosphine and 5-chloro-2-mercaptobenzothiazole (Aldrich, Merck) were used with no further purification. The IR spectra of the ligands and the complexes were recorded on the Perkin-Elmer spectrum *GX* FT—IR spectrophotometer in the range, 4000–370 cm^-1^ (using KBr pellets).

*Synthesis and crystallization of {[Ag(tpp)_3_Br] MeCN H_2_O} complex:* 0.094 g, AgBr (0.5 mmol),, 0.131 g triphenylphosphine (0.5 mmol), and 0.101 g 5-cloro-2-methylobenzimidazole (0.5 mmol) were suspended in 20 ml of toluene. The reaction mixture was refluxed for 3 h. The clear solution was filter off and concentrated. Yellow powder was collected and re-crystallized with 20 ml of hot CH_3_OH/CH_3_CN (1:1) solution. After slow evaporation of the clear solution derived at room temperature, yellow colored crystals were formed. Yield: 55%; m.p. 136–142 *^o^*C. Main IR peaks: (KBr, cm^-1^), 3066–2910 (C–H), 1582 (C–C), 1092 (P–CPh), 500–505 (P–CPh),.

### Refinement

Hydrogen atoms were located geometrically (*C*(methyl)-H 0.98 Å, C(ar)—H 0.95 Å) and refined as a riding model; the *U*_iso_ values of H atoms were set at 1.2 (1.5 for methyl groups) times *U*_eq_ of their carrier atom. The significant residual electron density observed in the voids was interpreted as the disordered water molecule which might come from the not dried solvent. The site occupation factors of disordered water molecule were constrained to sum up to unity; weak constraints were applied to the ADP's of these partially occupied atoms. The 12 reflections were probably obscured by the beamstop, and therefore the SQUEEZE procedure was not used.

### Crystal data

**Table:**

[AgBr(C_18_H_15_P)_3_]·C_2_H_3_N·H_2_O	*Z* = 2
*M**_r_* = 1033.66	*F*(000) = 1056
Triclinic, *P*1	*D*_x_ = 1.412 Mg m^−^^3^
*a* = 13.1894 (4) Å	Mo *K*α radiation, λ = 0.71073 Å
*b* = 13.7384 (5) Å	Cell parameters from 3472 reflections
*c* = 13.8299 (5) Å	θ = 3–22°
α = 84.103 (3)°	µ = 1.38 mm^−^^1^
β = 87.161 (3)°	*T* = 100 K
γ = 77.398 (3)°	Block, colourless
*V* = 2431.73 (14) Å^3^	0.3 × 0.3 × 0.2 mm

### Data collection

**Table:**

Agilent Xcalibur Sapphire2 diffractometer	9347 independent reflections
Radiation source: Enhance (Mo) X-ray Source	7605 reflections with *I* > 2σ(*I*)
graphite	*R*_int_ = 0.029
Detector resolution: 8.1929 pixels mm^-1^	θ_max_ = 26.0°, θ_min_ = 2.9°
ω scans	*h* = −12→16
Absorption correction: multi-scan (*CrysAlis PRO*; Agilent, 2010)	*k* = −16→16
*T*_min_ = 0.956, *T*_max_ = 1.000	*l* = −13→16
17990 measured reflections	

### Refinement

**Table:**

Refinement on *F*^2^	Primary atom site location: structure-invariant direct methods
Least-squares matrix: full	Secondary atom site location: difference Fourier map
*R*[*F*^2^ > 2σ(*F*^2^)] = 0.043	Hydrogen site location: inferred from neighbouring sites
*wR*(*F*^2^) = 0.102	H-atom parameters constrained
*S* = 1.04	*w* = 1/[σ^2^(*F*_o_^2^) + (0.0356*P*)^2^ + 3.8816*P*] where *P* = (*F*_o_^2^ + 2*F*_c_^2^)/3
9347 reflections	(Δ/σ)_max_ = 0.002
591 parameters	Δρ_max_ = 1.27 e Å^−^^3^
19 restraints	Δρ_min_ = −0.91 e Å^−^^3^

### Special details

**Table:**

Geometry. All s.u.'s (except the s.u. in the dihedral angle between two l.s. planes) are estimated using the full covariance matrix. The cell s.u.'s are taken into account individually in the estimation of s.u.'s in distances, angles and torsion angles; correlations between s.u.'s in cell parameters are only used when they are defined by crystal symmetry. An approximate (isotropic) treatment of cell s.u.'s is used for estimating s.u.'s involving l.s. planes.
Refinement. Refinement of *F*^2^ against ALL reflections. The weighted *R*-factor *wR* and goodness of fit *S* are based on *F*^2^, conventional *R*-factors *R* are based on *F*, with *F* set to zero for negative *F*^2^. The threshold expression of *F*^2^ > σ(*F*^2^) is used only for calculating *R*-factors(gt) *etc*. and is not relevant to the choice of reflections for refinement. *R*-factors based on *F*^2^ are statistically about twice as large as those based on *F*, and *R*- factors based on ALL data will be even larger.

### Fractional atomic coordinates and isotropic or equivalent isotropic displacement parameters (Å^2^)

**Table:**

	*x*	*y*	*z*	*U*_iso_*/*U*_eq_	Occ. (<1)
Br1	0.08140 (3)	0.25613 (3)	0.28539 (3)	0.03089 (12)	
Ag1	0.26631 (2)	0.31500 (2)	0.26939 (2)	0.01625 (9)	
P1	0.24106 (7)	0.49430 (7)	0.19144 (7)	0.0164 (2)	
C1	0.1628 (3)	0.5978 (3)	0.2541 (3)	0.0179 (8)	
P2	0.32797 (8)	0.29379 (7)	0.44479 (7)	0.0165 (2)	
C2	0.0579 (3)	0.5961 (3)	0.2728 (3)	0.0228 (9)	
H2A	0.0310	0.5417	0.2555	0.027*	
P3	0.35979 (7)	0.18064 (7)	0.16218 (7)	0.0158 (2)	
C3	−0.0067 (3)	0.6739 (3)	0.3166 (3)	0.0272 (10)	
H3A	−0.0780	0.6732	0.3288	0.033*	
C4	0.0323 (3)	0.7519 (3)	0.3423 (4)	0.0370 (12)	
H4A	−0.0122	0.8051	0.3723	0.044*	
C5	0.1364 (3)	0.7534 (3)	0.3247 (4)	0.0386 (12)	
H5A	0.1632	0.8075	0.3428	0.046*	
C6	0.2010 (3)	0.6760 (3)	0.2808 (3)	0.0272 (10)	
H6A	0.2724	0.6770	0.2690	0.033*	
C7	0.3675 (3)	0.5277 (3)	0.1747 (3)	0.0170 (8)	
C8	0.4364 (3)	0.4996 (3)	0.2509 (3)	0.0194 (8)	
H8A	0.4152	0.4662	0.3094	0.023*	
C9	0.5353 (3)	0.5195 (3)	0.2428 (3)	0.0231 (9)	
H9A	0.5814	0.4992	0.2953	0.028*	
C10	0.5667 (3)	0.5691 (3)	0.1581 (3)	0.0258 (9)	
H10A	0.6341	0.5835	0.1523	0.031*	
C11	0.4989 (3)	0.5974 (3)	0.0822 (3)	0.0304 (10)	
H11A	0.5203	0.6309	0.0237	0.036*	
C12	0.4004 (3)	0.5775 (3)	0.0903 (3)	0.0240 (9)	
H12A	0.3546	0.5980	0.0376	0.029*	
C13	0.1817 (3)	0.5224 (3)	0.0723 (3)	0.0171 (8)	
C14	0.1443 (3)	0.6208 (3)	0.0329 (3)	0.0210 (9)	
H14A	0.1522	0.6754	0.0666	0.025*	
C15	0.0955 (3)	0.6384 (3)	−0.0558 (3)	0.0228 (9)	
H15A	0.0698	0.7051	−0.0828	0.027*	
C16	0.0844 (3)	0.5586 (3)	−0.1051 (3)	0.0253 (9)	
H16A	0.0505	0.5710	−0.1656	0.030*	
C17	0.1221 (3)	0.4612 (3)	−0.0670 (3)	0.0258 (10)	
H17A	0.1151	0.4068	−0.1016	0.031*	
C18	0.1705 (3)	0.4428 (3)	0.0221 (3)	0.0206 (9)	
H18A	0.1959	0.3759	0.0488	0.025*	
C19	0.3179 (3)	0.1806 (3)	0.5236 (3)	0.0183 (8)	
C20	0.2246 (3)	0.1492 (3)	0.5265 (3)	0.0246 (9)	
H20A	0.1710	0.1831	0.4840	0.030*	
C21	0.2085 (3)	0.0692 (3)	0.5903 (3)	0.0289 (10)	
H21A	0.1443	0.0483	0.5913	0.035*	
C22	0.2862 (4)	0.0198 (3)	0.6526 (3)	0.0296 (10)	
H22A	0.2750	−0.0338	0.6980	0.036*	
C23	0.3795 (4)	0.0489 (3)	0.6481 (3)	0.0341 (11)	
H23A	0.4334	0.0139	0.6896	0.041*	
C24	0.3965 (3)	0.1285 (3)	0.5839 (3)	0.0283 (10)	
H24A	0.4618	0.1473	0.5813	0.034*	
C25	0.2550 (3)	0.3887 (3)	0.5198 (3)	0.0182 (8)	
C26	0.2735 (3)	0.3885 (3)	0.6183 (3)	0.0222 (9)	
H26A	0.3259	0.3376	0.6488	0.027*	
C27	0.2154 (3)	0.4622 (3)	0.6717 (3)	0.0250 (9)	
H27A	0.2285	0.4616	0.7388	0.030*	
C28	0.1388 (3)	0.5366 (3)	0.6286 (3)	0.0246 (9)	
H28A	0.1006	0.5879	0.6653	0.030*	
C29	0.1180 (3)	0.5361 (3)	0.5320 (3)	0.0264 (10)	
H29A	0.0642	0.5863	0.5024	0.032*	
C30	0.1754 (3)	0.4624 (3)	0.4781 (3)	0.0234 (9)	
H30A	0.1601	0.4621	0.4118	0.028*	
C31	0.4636 (3)	0.3000 (3)	0.4545 (3)	0.0185 (8)	
C32	0.4987 (3)	0.3671 (3)	0.5063 (3)	0.0221 (9)	
H32A	0.4501	0.4126	0.5419	0.027*	
C33	0.6032 (3)	0.3689 (3)	0.5069 (3)	0.0261 (9)	
H33A	0.6253	0.4157	0.5426	0.031*	
C34	0.6755 (3)	0.3032 (3)	0.4560 (3)	0.0264 (9)	
H34A	0.7472	0.3041	0.4569	0.032*	
C35	0.6420 (3)	0.2364 (3)	0.4038 (3)	0.0305 (10)	
H35A	0.6911	0.1911	0.3684	0.037*	
C36	0.5380 (3)	0.2350 (3)	0.4027 (3)	0.0261 (9)	
H36A	0.5163	0.1889	0.3658	0.031*	
C37	0.3544 (3)	0.0542 (3)	0.2132 (3)	0.0166 (8)	
C38	0.3362 (3)	0.0379 (3)	0.3137 (3)	0.0220 (9)	
H38A	0.3225	0.0929	0.3522	0.026*	
C39	0.3378 (3)	−0.0573 (3)	0.3569 (3)	0.0261 (9)	
H39A	0.3256	−0.0671	0.4251	0.031*	
C40	0.3570 (3)	−0.1386 (3)	0.3027 (3)	0.0247 (9)	
H40A	0.3603	−0.2042	0.3334	0.030*	
C41	0.3716 (3)	−0.1230 (3)	0.2026 (3)	0.0256 (9)	
H41A	0.3831	−0.1782	0.1644	0.031*	
C42	0.3694 (3)	−0.0279 (3)	0.1581 (3)	0.0225 (9)	
H42A	0.3780	−0.0182	0.0895	0.027*	
C43	0.3122 (3)	0.1823 (3)	0.0400 (3)	0.0178 (8)	
C44	0.2073 (3)	0.1817 (3)	0.0314 (3)	0.0256 (9)	
H44A	0.1640	0.1793	0.0882	0.031*	
C45	0.1657 (3)	0.1845 (3)	−0.0597 (3)	0.0325 (11)	
H45A	0.0942	0.1844	−0.0650	0.039*	
C46	0.2291 (4)	0.1875 (3)	−0.1427 (3)	0.0334 (11)	
H46A	0.2011	0.1881	−0.2048	0.040*	
C47	0.3321 (4)	0.1895 (3)	−0.1351 (3)	0.0295 (10)	
H47A	0.3747	0.1928	−0.1923	0.035*	
C48	0.3746 (3)	0.1868 (3)	−0.0439 (3)	0.0218 (9)	
H48A	0.4459	0.1879	−0.0393	0.026*	
C49	0.4984 (3)	0.1765 (3)	0.1417 (2)	0.0161 (8)	
C50	0.5350 (3)	0.2611 (3)	0.1562 (3)	0.0200 (8)	
H50A	0.4877	0.3177	0.1781	0.024*	
C51	0.6382 (3)	0.2656 (3)	0.1398 (3)	0.0247 (9)	
H51A	0.6612	0.3245	0.1502	0.030*	
C52	0.7076 (3)	0.1834 (3)	0.1080 (3)	0.0247 (9)	
H52A	0.7785	0.1861	0.0956	0.030*	
C53	0.6742 (3)	0.0973 (3)	0.0943 (3)	0.0243 (9)	
H53A	0.7223	0.0410	0.0730	0.029*	
C54	0.5703 (3)	0.0927 (3)	0.1115 (3)	0.0217 (9)	
H54A	0.5479	0.0331	0.1028	0.026*	
C2S	0.0734 (4)	0.9380 (4)	0.1057 (5)	0.0554 (15)	
C3S	0.0522 (6)	1.0083 (5)	0.1777 (5)	0.088 (2)	
H56A	0.0130	1.0728	0.1486	0.132*	
H56B	0.1179	1.0173	0.2018	0.132*	
H56C	0.0113	0.9830	0.2317	0.132*	
N1S	0.0872 (4)	0.8816 (4)	0.0475 (4)	0.0598 (13)	
O58R	0.9214 (6)	0.1285 (6)	0.4412 (7)	0.040 (2)	0.404 (4)
O68A	0.9014 (9)	0.1377 (10)	0.3565 (10)	0.045 (4)	0.279 (9)
O68B	1.0064 (10)	0.0597 (10)	0.4154 (9)	0.065 (5)	0.317 (9)

### Atomic displacement parameters (Å^2^)

**Table:**

	*U*^11^	*U*^22^	*U*^33^	*U*^12^	*U*^13^	*U*^23^
Br1	0.0208 (2)	0.0314 (2)	0.0393 (3)	−0.00299 (18)	−0.00427 (18)	−0.0019 (2)
Ag1	0.02006 (16)	0.01514 (15)	0.01274 (15)	−0.00167 (11)	−0.00147 (11)	−0.00159 (11)
P1	0.0193 (5)	0.0153 (5)	0.0137 (5)	−0.0020 (4)	−0.0037 (4)	0.0004 (4)
C1	0.023 (2)	0.0165 (19)	0.0130 (19)	−0.0017 (16)	−0.0028 (15)	0.0029 (15)
P2	0.0221 (5)	0.0155 (5)	0.0113 (5)	−0.0025 (4)	−0.0024 (4)	−0.0008 (4)
C2	0.025 (2)	0.023 (2)	0.019 (2)	−0.0037 (17)	−0.0062 (16)	0.0015 (17)
P3	0.0201 (5)	0.0144 (5)	0.0119 (5)	−0.0012 (4)	−0.0005 (4)	−0.0016 (4)
C3	0.018 (2)	0.029 (2)	0.032 (2)	−0.0004 (18)	0.0012 (17)	−0.0020 (19)
C4	0.030 (3)	0.025 (2)	0.054 (3)	0.001 (2)	0.012 (2)	−0.013 (2)
C5	0.026 (2)	0.027 (2)	0.065 (3)	−0.007 (2)	0.013 (2)	−0.021 (2)
C6	0.022 (2)	0.024 (2)	0.037 (3)	−0.0069 (18)	0.0041 (18)	−0.0095 (19)
C7	0.020 (2)	0.0133 (18)	0.018 (2)	−0.0030 (15)	−0.0021 (15)	−0.0018 (15)
C8	0.028 (2)	0.0146 (19)	0.015 (2)	−0.0030 (16)	−0.0025 (16)	0.0005 (15)
C9	0.021 (2)	0.027 (2)	0.021 (2)	−0.0029 (17)	−0.0055 (16)	−0.0040 (17)
C10	0.023 (2)	0.032 (2)	0.024 (2)	−0.0083 (18)	−0.0012 (17)	−0.0002 (18)
C11	0.028 (2)	0.040 (3)	0.022 (2)	−0.010 (2)	0.0001 (18)	0.0071 (19)
C12	0.024 (2)	0.027 (2)	0.018 (2)	−0.0037 (18)	−0.0039 (16)	0.0054 (17)
C13	0.0165 (19)	0.022 (2)	0.0125 (19)	−0.0034 (16)	−0.0029 (14)	0.0013 (15)
C14	0.026 (2)	0.019 (2)	0.018 (2)	−0.0040 (17)	−0.0041 (16)	0.0019 (16)
C15	0.021 (2)	0.025 (2)	0.020 (2)	−0.0030 (17)	−0.0024 (16)	0.0040 (17)
C16	0.023 (2)	0.038 (2)	0.013 (2)	−0.0037 (19)	−0.0024 (16)	0.0014 (18)
C17	0.030 (2)	0.030 (2)	0.017 (2)	−0.0026 (19)	−0.0012 (17)	−0.0079 (18)
C18	0.021 (2)	0.020 (2)	0.018 (2)	−0.0006 (16)	0.0000 (15)	0.0002 (16)
C19	0.030 (2)	0.0160 (19)	0.0085 (18)	−0.0028 (17)	−0.0011 (15)	−0.0027 (15)
C20	0.025 (2)	0.023 (2)	0.023 (2)	−0.0004 (18)	−0.0013 (17)	−0.0002 (17)
C21	0.032 (2)	0.020 (2)	0.034 (3)	−0.0057 (19)	0.0101 (19)	−0.0040 (19)
C22	0.048 (3)	0.017 (2)	0.022 (2)	−0.004 (2)	0.0076 (19)	0.0008 (17)
C23	0.044 (3)	0.028 (2)	0.025 (2)	0.001 (2)	−0.010 (2)	0.0062 (19)
C24	0.033 (2)	0.026 (2)	0.025 (2)	−0.0066 (19)	−0.0093 (18)	0.0041 (18)
C25	0.022 (2)	0.019 (2)	0.015 (2)	−0.0055 (16)	0.0005 (15)	−0.0033 (16)
C26	0.022 (2)	0.027 (2)	0.017 (2)	−0.0047 (17)	−0.0030 (16)	−0.0031 (17)
C27	0.026 (2)	0.038 (2)	0.015 (2)	−0.0125 (19)	0.0029 (16)	−0.0095 (18)
C28	0.024 (2)	0.022 (2)	0.029 (2)	−0.0067 (18)	0.0065 (17)	−0.0083 (18)
C29	0.026 (2)	0.023 (2)	0.027 (2)	0.0004 (18)	0.0018 (17)	−0.0005 (18)
C30	0.027 (2)	0.022 (2)	0.021 (2)	−0.0048 (18)	−0.0026 (16)	0.0017 (17)
C31	0.026 (2)	0.0169 (19)	0.0117 (19)	−0.0041 (16)	−0.0021 (15)	0.0034 (15)
C32	0.026 (2)	0.018 (2)	0.021 (2)	−0.0013 (17)	0.0006 (16)	−0.0037 (16)
C33	0.029 (2)	0.024 (2)	0.027 (2)	−0.0078 (19)	−0.0010 (18)	−0.0049 (18)
C34	0.023 (2)	0.029 (2)	0.026 (2)	−0.0055 (18)	−0.0022 (17)	0.0050 (18)
C35	0.030 (2)	0.030 (2)	0.028 (2)	−0.002 (2)	0.0060 (18)	−0.0058 (19)
C36	0.031 (2)	0.028 (2)	0.020 (2)	−0.0050 (19)	−0.0024 (17)	−0.0082 (18)
C37	0.0150 (19)	0.0179 (19)	0.016 (2)	−0.0011 (15)	−0.0005 (14)	−0.0007 (15)
C38	0.027 (2)	0.021 (2)	0.018 (2)	−0.0038 (17)	0.0011 (16)	−0.0053 (17)
C39	0.033 (2)	0.025 (2)	0.019 (2)	−0.0053 (19)	−0.0015 (17)	0.0040 (17)
C40	0.025 (2)	0.017 (2)	0.033 (2)	−0.0080 (17)	−0.0021 (17)	0.0042 (18)
C41	0.029 (2)	0.020 (2)	0.030 (2)	−0.0077 (18)	0.0012 (18)	−0.0086 (18)
C42	0.027 (2)	0.020 (2)	0.020 (2)	−0.0047 (17)	0.0022 (16)	−0.0032 (17)
C43	0.026 (2)	0.0122 (18)	0.015 (2)	−0.0016 (16)	−0.0041 (15)	−0.0014 (15)
C44	0.026 (2)	0.025 (2)	0.025 (2)	−0.0022 (18)	0.0002 (17)	−0.0044 (18)
C45	0.033 (2)	0.028 (2)	0.034 (3)	0.005 (2)	−0.015 (2)	−0.011 (2)
C46	0.048 (3)	0.025 (2)	0.025 (2)	0.007 (2)	−0.017 (2)	−0.0104 (19)
C47	0.045 (3)	0.024 (2)	0.015 (2)	0.001 (2)	−0.0027 (18)	−0.0007 (17)
C48	0.028 (2)	0.017 (2)	0.017 (2)	0.0029 (17)	−0.0007 (16)	−0.0019 (16)
C49	0.0197 (19)	0.0189 (19)	0.0083 (18)	−0.0017 (16)	−0.0012 (14)	0.0005 (15)
C50	0.026 (2)	0.019 (2)	0.014 (2)	−0.0034 (17)	−0.0013 (15)	0.0010 (16)
C51	0.030 (2)	0.026 (2)	0.019 (2)	−0.0105 (18)	0.0012 (17)	−0.0001 (17)
C52	0.020 (2)	0.033 (2)	0.021 (2)	−0.0053 (18)	0.0001 (16)	0.0026 (18)
C53	0.021 (2)	0.026 (2)	0.022 (2)	0.0009 (18)	0.0019 (16)	0.0020 (17)
C54	0.028 (2)	0.016 (2)	0.021 (2)	−0.0027 (17)	−0.0007 (16)	−0.0028 (16)
C2S	0.052 (3)	0.041 (3)	0.075 (4)	−0.017 (3)	0.007 (3)	−0.003 (3)
C3S	0.133 (7)	0.056 (4)	0.084 (5)	−0.037 (4)	0.021 (5)	−0.021 (4)
N1S	0.057 (3)	0.047 (3)	0.073 (4)	−0.004 (2)	0.014 (3)	−0.014 (3)
O58R	0.026 (4)	0.040 (4)	0.057 (5)	−0.015 (3)	0.016 (4)	−0.003 (4)
O68A	0.034 (6)	0.066 (8)	0.050 (8)	−0.036 (5)	0.014 (5)	−0.025 (6)
O68B	0.078 (8)	0.071 (8)	0.063 (7)	−0.052 (6)	0.008 (6)	−0.009 (6)

### Geometric parameters (Å, °)

**Table:**

Br1—Ag1	2.7242 (5)	C26—C27	1.384 (5)
Ag1—P1	2.5441 (10)	C26—H26A	0.9500
Ag1—P3	2.5523 (9)	C27—C28	1.380 (5)
Ag1—P2	2.5647 (10)	C27—H27A	0.9500
P1—C7	1.822 (4)	C28—C29	1.378 (6)
P1—C13	1.828 (4)	C28—H28A	0.9500
P1—C1	1.833 (4)	C29—C30	1.385 (5)
C1—C6	1.371 (5)	C29—H29A	0.9500
C1—C2	1.400 (5)	C30—H30A	0.9500
P2—C31	1.822 (4)	C31—C32	1.389 (5)
P2—C25	1.826 (4)	C31—C36	1.398 (5)
P2—C19	1.831 (4)	C32—C33	1.384 (6)
C2—C3	1.386 (5)	C32—H32A	0.9500
C2—H2A	0.9500	C33—C34	1.381 (5)
P3—C37	1.821 (4)	C33—H33A	0.9500
P3—C49	1.826 (4)	C34—C35	1.379 (6)
P3—C43	1.828 (4)	C34—H34A	0.9500
C3—C4	1.368 (6)	C35—C36	1.378 (6)
C3—H3A	0.9500	C35—H35A	0.9500
C4—C5	1.386 (6)	C36—H36A	0.9500
C4—H4A	0.9500	C37—C42	1.399 (5)
C5—C6	1.384 (5)	C37—C38	1.402 (5)
C5—H5A	0.9500	C38—C39	1.377 (6)
C6—H6A	0.9500	C38—H38A	0.9500
C7—C12	1.390 (5)	C39—C40	1.379 (6)
C7—C8	1.394 (5)	C39—H39A	0.9500
C8—C9	1.387 (6)	C40—C41	1.390 (6)
C8—H8A	0.9500	C40—H40A	0.9500
C9—C10	1.385 (6)	C41—C42	1.380 (6)
C9—H9A	0.9500	C41—H41A	0.9500
C10—C11	1.383 (5)	C42—H42A	0.9500
C10—H10A	0.9500	C43—C48	1.393 (5)
C11—C12	1.382 (6)	C43—C44	1.397 (5)
C11—H11A	0.9500	C44—C45	1.393 (6)
C12—H12A	0.9500	C44—H44A	0.9500
C13—C18	1.391 (5)	C45—C46	1.388 (6)
C13—C14	1.398 (5)	C45—H45A	0.9500
C14—C15	1.388 (5)	C46—C47	1.375 (6)
C14—H14A	0.9500	C46—H46A	0.9500
C15—C16	1.386 (6)	C47—C48	1.399 (5)
C15—H15A	0.9500	C47—H47A	0.9500
C16—C17	1.381 (6)	C48—H48A	0.9500
C16—H16A	0.9500	C49—C50	1.388 (5)
C17—C18	1.391 (5)	C49—C54	1.408 (5)
C17—H17A	0.9500	C50—C51	1.382 (6)
C18—H18A	0.9500	C50—H50A	0.9500
C19—C24	1.386 (5)	C51—C52	1.385 (5)
C19—C20	1.388 (6)	C51—H51A	0.9500
C20—C21	1.384 (6)	C52—C53	1.382 (6)
C20—H20A	0.9500	C52—H52A	0.9500
C21—C22	1.385 (6)	C53—C54	1.393 (6)
C21—H21A	0.9500	C53—H53A	0.9500
C22—C23	1.371 (7)	C54—H54A	0.9500
C22—H22A	0.9500	C2S—N1S	1.155 (7)
C23—C24	1.385 (6)	C2S—C3S	1.433 (8)
C23—H23A	0.9500	C3S—H56A	0.9800
C24—H24A	0.9500	C3S—H56B	0.9800
C25—C30	1.392 (5)	C3S—H56C	0.9800
C25—C26	1.395 (5)		
			
P1—Ag1—P3	115.30 (3)	C30—C25—P2	118.8 (3)
P1—Ag1—P2	113.32 (3)	C26—C25—P2	122.7 (3)
P3—Ag1—P2	115.22 (3)	C27—C26—C25	120.2 (4)
P1—Ag1—Br1	110.24 (3)	C27—C26—H26A	119.9
P3—Ag1—Br1	96.64 (3)	C25—C26—H26A	119.9
P2—Ag1—Br1	104.01 (3)	C28—C27—C26	120.7 (4)
C7—P1—C13	105.64 (17)	C28—C27—H27A	119.7
C7—P1—C1	103.23 (17)	C26—C27—H27A	119.7
C13—P1—C1	99.59 (16)	C29—C28—C27	119.7 (4)
C7—P1—Ag1	108.81 (12)	C29—C28—H28A	120.1
C13—P1—Ag1	117.50 (13)	C27—C28—H28A	120.1
C1—P1—Ag1	120.33 (12)	C28—C29—C30	119.9 (4)
C6—C1—C2	119.5 (3)	C28—C29—H29A	120.0
C6—C1—P1	123.7 (3)	C30—C29—H29A	120.0
C2—C1—P1	116.8 (3)	C29—C30—C25	121.0 (4)
C31—P2—C25	105.08 (17)	C29—C30—H30A	119.5
C31—P2—C19	102.96 (17)	C25—C30—H30A	119.5
C25—P2—C19	99.63 (17)	C32—C31—C36	117.3 (4)
C31—P2—Ag1	113.94 (12)	C32—C31—P2	125.2 (3)
C25—P2—Ag1	113.50 (12)	C36—C31—P2	117.5 (3)
C19—P2—Ag1	119.76 (12)	C33—C32—C31	121.3 (4)
C3—C2—C1	119.9 (4)	C33—C32—H32A	119.4
C3—C2—H2A	120.1	C31—C32—H32A	119.4
C1—C2—H2A	120.1	C34—C33—C32	120.5 (4)
C37—P3—C49	104.13 (16)	C34—C33—H33A	119.8
C37—P3—C43	101.50 (17)	C32—C33—H33A	119.8
C49—P3—C43	103.68 (17)	C35—C34—C33	119.1 (4)
C37—P3—Ag1	112.95 (12)	C35—C34—H34A	120.5
C49—P3—Ag1	114.55 (12)	C33—C34—H34A	120.5
C43—P3—Ag1	118.25 (11)	C36—C35—C34	120.5 (4)
C4—C3—C2	120.1 (4)	C36—C35—H35A	119.7
C4—C3—H3A	120.0	C34—C35—H35A	119.7
C2—C3—H3A	120.0	C35—C36—C31	121.4 (4)
C3—C4—C5	120.3 (4)	C35—C36—H36A	119.3
C3—C4—H4A	119.8	C31—C36—H36A	119.3
C5—C4—H4A	119.8	C42—C37—C38	118.1 (4)
C6—C5—C4	119.8 (4)	C42—C37—P3	123.7 (3)
C6—C5—H5A	120.1	C38—C37—P3	118.2 (3)
C4—C5—H5A	120.1	C39—C38—C37	120.5 (4)
C1—C6—C5	120.5 (4)	C39—C38—H38A	119.7
C1—C6—H6A	119.8	C37—C38—H38A	119.7
C5—C6—H6A	119.8	C38—C39—C40	121.0 (4)
C12—C7—C8	118.0 (4)	C38—C39—H39A	119.5
C12—C7—P1	124.3 (3)	C40—C39—H39A	119.5
C8—C7—P1	117.7 (3)	C39—C40—C41	119.0 (4)
C9—C8—C7	121.3 (4)	C39—C40—H40A	120.5
C9—C8—H8A	119.3	C41—C40—H40A	120.5
C7—C8—H8A	119.3	C42—C41—C40	120.6 (4)
C10—C9—C8	119.9 (4)	C42—C41—H41A	119.7
C10—C9—H9A	120.1	C40—C41—H41A	119.7
C8—C9—H9A	120.1	C41—C42—C37	120.6 (4)
C11—C10—C9	119.3 (4)	C41—C42—H42A	119.7
C11—C10—H10A	120.4	C37—C42—H42A	119.7
C9—C10—H10A	120.4	C48—C43—C44	119.1 (3)
C12—C11—C10	120.8 (4)	C48—C43—P3	123.2 (3)
C12—C11—H11A	119.6	C44—C43—P3	117.6 (3)
C10—C11—H11A	119.6	C45—C44—C43	120.5 (4)
C11—C12—C7	120.8 (4)	C45—C44—H44A	119.8
C11—C12—H12A	119.6	C43—C44—H44A	119.8
C7—C12—H12A	119.6	C46—C45—C44	119.8 (4)
C18—C13—C14	119.8 (3)	C46—C45—H45A	120.1
C18—C13—P1	118.4 (3)	C44—C45—H45A	120.1
C14—C13—P1	121.7 (3)	C47—C46—C45	120.1 (4)
C15—C14—C13	119.6 (4)	C47—C46—H46A	119.9
C15—C14—H14A	120.2	C45—C46—H46A	119.9
C13—C14—H14A	120.2	C46—C47—C48	120.5 (4)
C16—C15—C14	120.1 (4)	C46—C47—H47A	119.8
C16—C15—H15A	120.0	C48—C47—H47A	119.8
C14—C15—H15A	120.0	C43—C48—C47	119.9 (4)
C17—C16—C15	120.5 (4)	C43—C48—H48A	120.0
C17—C16—H16A	119.7	C47—C48—H48A	120.0
C15—C16—H16A	119.7	C50—C49—C54	118.1 (3)
C16—C17—C18	119.8 (4)	C50—C49—P3	118.1 (3)
C16—C17—H17A	120.1	C54—C49—P3	123.8 (3)
C18—C17—H17A	120.1	C51—C50—C49	122.0 (4)
C13—C18—C17	120.1 (4)	C51—C50—H50A	119.0
C13—C18—H18A	120.0	C49—C50—H50A	119.0
C17—C18—H18A	120.0	C50—C51—C52	119.3 (4)
C24—C19—C20	118.9 (4)	C50—C51—H51A	120.4
C24—C19—P2	123.3 (3)	C52—C51—H51A	120.4
C20—C19—P2	117.8 (3)	C53—C52—C51	120.2 (4)
C21—C20—C19	120.9 (4)	C53—C52—H52A	119.9
C21—C20—H20A	119.5	C51—C52—H52A	119.9
C19—C20—H20A	119.5	C52—C53—C54	120.4 (4)
C20—C21—C22	119.7 (4)	C52—C53—H53A	119.8
C20—C21—H21A	120.1	C54—C53—H53A	119.8
C22—C21—H21A	120.1	C53—C54—C49	120.0 (4)
C23—C22—C21	119.4 (4)	C53—C54—H54A	120.0
C23—C22—H22A	120.3	C49—C54—H54A	120.0
C21—C22—H22A	120.3	N1S—C2S—C3S	177.9 (7)
C22—C23—C24	121.2 (4)	C2S—C3S—H56A	109.5
C22—C23—H23A	119.4	C2S—C3S—H56B	109.5
C24—C23—H23A	119.4	H56A—C3S—H56B	109.5
C23—C24—C19	119.8 (4)	C2S—C3S—H56C	109.5
C23—C24—H24A	120.1	H56A—C3S—H56C	109.5
C19—C24—H24A	120.1	H56B—C3S—H56C	109.5
C30—C25—C26	118.5 (3)		
			
P3—Ag1—P1—C7	−68.14 (13)	C19—C20—C21—C22	0.2 (6)
P2—Ag1—P1—C7	67.66 (13)	C20—C21—C22—C23	−2.0 (6)
Br1—Ag1—P1—C7	−176.23 (12)	C21—C22—C23—C24	1.6 (6)
P3—Ag1—P1—C13	51.75 (14)	C22—C23—C24—C19	0.6 (7)
P2—Ag1—P1—C13	−172.45 (13)	C20—C19—C24—C23	−2.4 (6)
Br1—Ag1—P1—C13	−56.34 (14)	P2—C19—C24—C23	174.0 (3)
P3—Ag1—P1—C1	173.22 (14)	C31—P2—C25—C30	124.9 (3)
P2—Ag1—P1—C1	−50.98 (15)	C19—P2—C25—C30	−128.8 (3)
Br1—Ag1—P1—C1	65.13 (15)	Ag1—P2—C25—C30	−0.2 (4)
C7—P1—C1—C6	−1.8 (4)	C31—P2—C25—C26	−57.4 (4)
C13—P1—C1—C6	−110.5 (4)	C19—P2—C25—C26	48.9 (4)
Ag1—P1—C1—C6	119.6 (3)	Ag1—P2—C25—C26	177.5 (3)
C7—P1—C1—C2	176.6 (3)	C30—C25—C26—C27	−2.1 (6)
C13—P1—C1—C2	67.9 (3)	P2—C25—C26—C27	−179.8 (3)
Ag1—P1—C1—C2	−61.9 (3)	C25—C26—C27—C28	0.1 (6)
P1—Ag1—P2—C31	−73.63 (14)	C26—C27—C28—C29	1.7 (6)
P3—Ag1—P2—C31	62.20 (14)	C27—C28—C29—C30	−1.4 (6)
Br1—Ag1—P2—C31	166.63 (13)	C28—C29—C30—C25	−0.6 (7)
P1—Ag1—P2—C25	46.60 (15)	C26—C25—C30—C29	2.3 (6)
P3—Ag1—P2—C25	−177.56 (14)	P2—C25—C30—C29	−179.9 (3)
Br1—Ag1—P2—C25	−73.13 (14)	C25—P2—C31—C32	−2.2 (4)
P1—Ag1—P2—C19	163.91 (14)	C19—P2—C31—C32	−106.0 (3)
P3—Ag1—P2—C19	−60.25 (15)	Ag1—P2—C31—C32	122.7 (3)
Br1—Ag1—P2—C19	44.18 (14)	C25—P2—C31—C36	−179.4 (3)
C6—C1—C2—C3	0.9 (6)	C19—P2—C31—C36	76.8 (3)
P1—C1—C2—C3	−177.5 (3)	Ag1—P2—C31—C36	−54.5 (3)
P1—Ag1—P3—C37	−169.47 (13)	C36—C31—C32—C33	−0.4 (6)
P2—Ag1—P3—C37	55.58 (13)	P2—C31—C32—C33	−177.6 (3)
Br1—Ag1—P3—C37	−53.34 (13)	C31—C32—C33—C34	−0.3 (6)
P1—Ag1—P3—C49	71.52 (13)	C32—C33—C34—C35	0.6 (6)
P2—Ag1—P3—C49	−63.44 (13)	C33—C34—C35—C36	−0.2 (6)
Br1—Ag1—P3—C49	−172.36 (12)	C34—C35—C36—C31	−0.6 (6)
P1—Ag1—P3—C43	−51.21 (16)	C32—C31—C36—C35	0.9 (6)
P2—Ag1—P3—C43	173.84 (15)	P2—C31—C36—C35	178.3 (3)
Br1—Ag1—P3—C43	64.92 (15)	C49—P3—C37—C42	−76.0 (3)
C1—C2—C3—C4	−0.5 (6)	C43—P3—C37—C42	31.4 (4)
C2—C3—C4—C5	0.0 (7)	Ag1—P3—C37—C42	159.1 (3)
C3—C4—C5—C6	0.2 (8)	C49—P3—C37—C38	102.8 (3)
C2—C1—C6—C5	−0.8 (6)	C43—P3—C37—C38	−149.7 (3)
P1—C1—C6—C5	177.6 (3)	Ag1—P3—C37—C38	−22.1 (3)
C4—C5—C6—C1	0.2 (7)	C42—C37—C38—C39	3.0 (6)
C13—P1—C7—C12	8.2 (4)	P3—C37—C38—C39	−175.9 (3)
C1—P1—C7—C12	−95.9 (3)	C37—C38—C39—C40	−0.3 (6)
Ag1—P1—C7—C12	135.2 (3)	C38—C39—C40—C41	−2.0 (6)
C13—P1—C7—C8	−170.4 (3)	C39—C40—C41—C42	1.6 (6)
C1—P1—C7—C8	85.5 (3)	C40—C41—C42—C37	1.2 (6)
Ag1—P1—C7—C8	−43.4 (3)	C38—C37—C42—C41	−3.5 (6)
C12—C7—C8—C9	−0.6 (5)	P3—C37—C42—C41	175.4 (3)
P1—C7—C8—C9	178.0 (3)	C37—P3—C43—C48	−111.9 (3)
C7—C8—C9—C10	0.6 (6)	C49—P3—C43—C48	−4.1 (4)
C8—C9—C10—C11	−0.6 (6)	Ag1—P3—C43—C48	124.0 (3)
C9—C10—C11—C12	0.5 (7)	C37—P3—C43—C44	69.8 (3)
C10—C11—C12—C7	−0.6 (7)	C49—P3—C43—C44	177.7 (3)
C8—C7—C12—C11	0.6 (6)	Ag1—P3—C43—C44	−54.3 (3)
P1—C7—C12—C11	−178.0 (3)	C48—C43—C44—C45	0.6 (6)
C7—P1—C13—C18	110.1 (3)	P3—C43—C44—C45	179.0 (3)
C1—P1—C13—C18	−143.1 (3)	C43—C44—C45—C46	0.3 (6)
Ag1—P1—C13—C18	−11.4 (4)	C44—C45—C46—C47	−1.2 (6)
C7—P1—C13—C14	−72.4 (3)	C45—C46—C47—C48	1.2 (6)
C1—P1—C13—C14	34.4 (4)	C44—C43—C48—C47	−0.6 (6)
Ag1—P1—C13—C14	166.1 (3)	P3—C43—C48—C47	−178.9 (3)
C18—C13—C14—C15	0.4 (6)	C46—C47—C48—C43	−0.3 (6)
P1—C13—C14—C15	−177.1 (3)	C37—P3—C49—C50	−144.3 (3)
C13—C14—C15—C16	−0.2 (6)	C43—P3—C49—C50	109.8 (3)
C14—C15—C16—C17	−0.5 (6)	Ag1—P3—C49—C50	−20.5 (3)
C15—C16—C17—C18	0.9 (6)	C37—P3—C49—C54	36.1 (3)
C14—C13—C18—C17	0.0 (6)	C43—P3—C49—C54	−69.8 (3)
P1—C13—C18—C17	177.5 (3)	Ag1—P3—C49—C54	159.9 (3)
C16—C17—C18—C13	−0.6 (6)	C54—C49—C50—C51	1.5 (5)
C31—P2—C19—C24	8.7 (4)	P3—C49—C50—C51	−178.1 (3)
C25—P2—C19—C24	−99.3 (3)	C49—C50—C51—C52	−0.1 (6)
Ag1—P2—C19—C24	136.4 (3)	C50—C51—C52—C53	−0.9 (6)
C31—P2—C19—C20	−174.9 (3)	C51—C52—C53—C54	0.5 (6)
C25—P2—C19—C20	77.1 (3)	C52—C53—C54—C49	0.9 (6)
Ag1—P2—C19—C20	−47.2 (3)	C50—C49—C54—C53	−1.8 (5)
C24—C19—C20—C21	2.0 (6)	P3—C49—C54—C53	177.7 (3)
P2—C19—C20—C21	−174.6 (3)		
